# Mini incision open pyeloplasty - Improvement in patient outcome

**DOI:** 10.1590/S1677-5538.IBJU.2014.0024

**Published:** 2015

**Authors:** Vishwajeet Singh, Manish Garg, Pradeep Sharma, Rahul Janak Sinha, Manoj Kumar

**Affiliations:** 1 Department of Urology, King George Medical University, Lucknow, India

**Keywords:** Laparoscopy, Minimally Invasive Surgical Procedures, Postoperative Period

## Abstract

**Purpose:**

To assess the subjective and objective outcomes of mini-incision dismembered Anderson-Hynes pyeloplasty in the treatment of primary ureteropelvic junction obstruction (UPJO).

**Materials and Methods:**

Between January 2008 to January 2013, Anderson-Hynes pyeloplasty was performed in 71 patients diagnosed with primary UPJO. Small subcostal muscle splitting incision was used in all cases. Sixteen patients with renal calculi underwent concomitant pyelolithotomy. Subjective outcome was assessed using visual pain analogue score (VAS). For objective assessment, the improvement in differential renal function (DRF) and radio-tracer wash out time (T1/2) on Tc-99m DTPA scan and decrease in hydronephrosis (HDN) on renal ultrasound (USG) and urography (IVU) were assessed.

**Results:**

Mean incision length was 5.2 cm. The average operating time and postoperative hospital stay was 63 (52-124) minutes and 2.5 (2–6) days respectively. Concomitant renal calculi were successfully removed in all the patients. Overall complication rates were 8.4% and overall success rate was 98.6% at median follow-up of 16 months. There was significant improvement in pain score (p=0.0001) and significant decrease in HDN after the procedure. While preoperative mean T1/2 was 26.7±6.4 minutes, postoperative half-time decreased to 7.8±4.2 minutes at 6 months and to 6.7±3.3 minutes at 1 year. Mean pre-operative DRF was 26.45% and it was 31.38% and 33.19% at 6 months and 1 year respectively.

**Conclusions:**

Mini-incision pyeloplasty is a safe and effective technique with combined advantage of high success rates of standard open pyeloplasty with decreased morbidity of laparoscopic approach. Excellent functional and objective outcomes can be achieved without extra technical difficulty.

## INTRODUCTION

In the current era, the treatment options of ureteropelvic junction obstruction (UPJO) vary from standard open pyeloplasty to various minimally invasive approaches. Traditionally, open pyeloplasty is considered as the reference standard for UPJO with success rates of 90% to 100%, against which all other treatment options are usually compared (1, 2). In standard open pyeloplasty large muscle cutting incision (about 20 to 24 cm) is generally used, which is the major cause of postoperative morbidity and pain (2, 3).

With the advancement of technology, various endoscopic and laparoscopic procedures were proposed for the treatment of UPJO. The laparoscopic techniques are increasingly employed for urological diseases and can be done via both transperitoneal or retroperitoneal approaches (4, 5) while procedures of antegrade and retrograde endopyelotomies, although minimally invasive, tend to have lower success rates with significant risk of bleeding (6). Though laparoscopic pyeloplasty is gaining popularity in urological practice because of less morbidity as compared to open standard pyeloplasty, laparoscopy is technically more difficult and challenging especially suturing. The laparoscopic pyeloplasty is also time consuming especially in inexperienced hands. Longer and steep learning curve is the main limiting factor of the procedure. In addition, laparoscopy requires specialized and expensive equipment thus increasing the overall cost of procedure. The technique is even more difficult while employing the retroperitoneoscopic laparoscopic approach (7, 8).

There are few reports which used small mini-incision for urological procedures for ureterolithotomy, nephrectomy or pyeloplasty which is defined as less than 10 cm in adults in different series (9–12). We contend that by refining the technique of open pyeloplasty to a much smaller incision, morbidity of the standard open technique can be minimized without compromising the success rates. Due to sheer advantages of mini-incision pyeloplasty (MIP), we decided to describe our experience and results of MIP in the present series.

## MATERIALS AND METHODS

A total of 71 consecutive patients were enrolled in this study from January 2008 to January 2013. The institutional ethical approval was obtained and it was in accordance with the declaration of Helsinki. All patients with BMI < 30kg/m^2^ with primary UPJO were included in the study.

The patients with renal function of less than 15%, uncorrected coagulopathy, vertebro-spinal deformity, and the presence of cardiopulmonary or respiratory compromise were excluded from study. Apart from the clinical history, physical examination and blood investigations, imaging studies including ultrasonography, contrast enhanced computerized tomography (CECT KUB) or intravenous pyelography (IVP) were done. Diuretic Tc-99m DTPA renal scan was done in all cases to assess the drainage pattern of the kidney, radio-tracer wash out time and differential renal function (DRF). The chest X-ray P.A view, electrocardiogram and pulmonary function test were performed to assess fitness for anesthesia. Urinary tract infection was treated preoperatively considering antibiotic sensitivity. Informed written consent was obtained prior to surgical intervention. Sixteen patients were found to have associated renal pelvic or calyceal calculi at diagnosis and two patients had horse-shoe kidney with unilateral UPJO.

### Open technique

**Figure 1:**
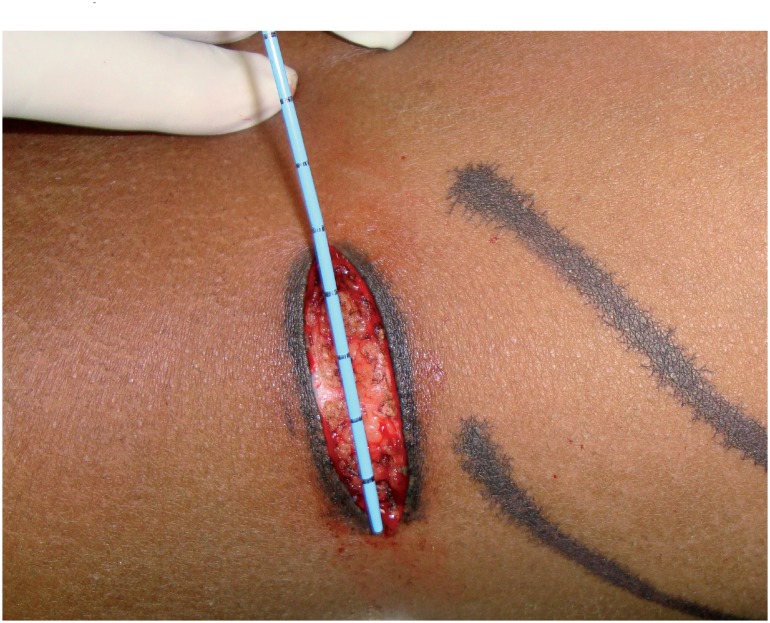
Subcostal muscle splitting incision (mean length 5.2 cm).

**Figure 2:**
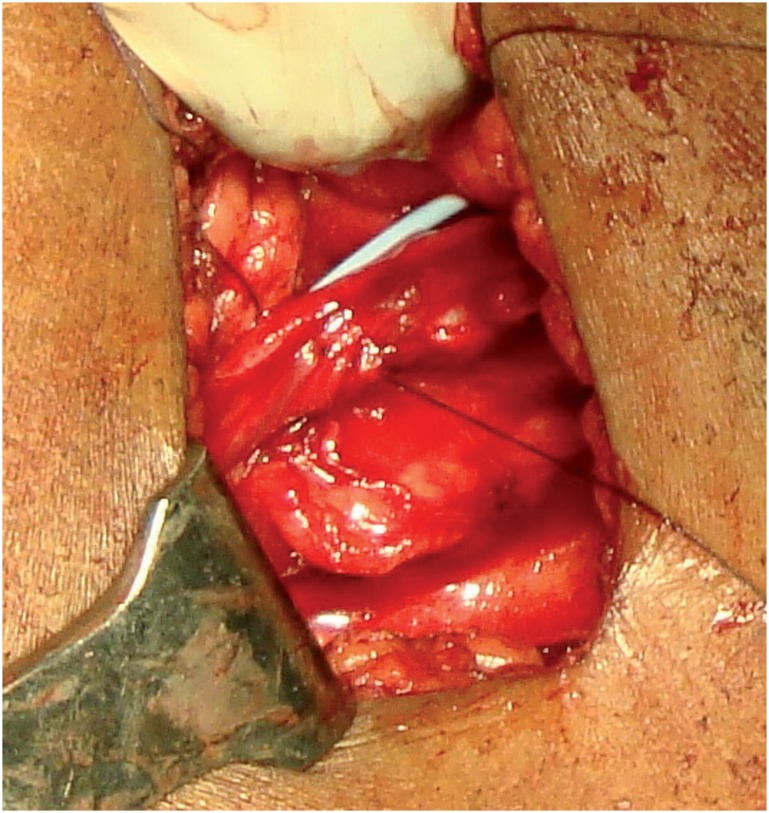
Antegrade DJ stent placement.

**Figure 3:**
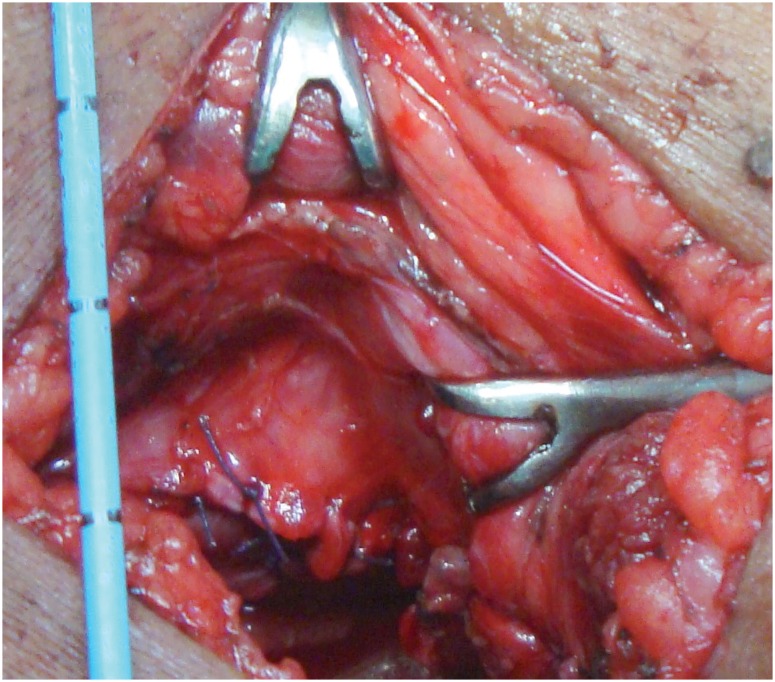
Water-tight anastomosis.

All operations were done by a single urologist. For open pyeloplasty lateral decubitus position was used and lumber subcostal muscle splitting incision was made with incision length of less than 8 cm (Figures 1 and 2). The abdominal muscles were separated and the peritoneum was pushed back. Gerota's fascia was opened and pelvis approached by identifying ureter over psoas muscle and by dissecting the ureter proximally. All dissections were performed with help of long retractors and instruments. The redundant part of pelvis was excised. After adequate spatulation of ureter, the anastomosis was performed by 4-0/50 vicryl continuous suture. Anderson Hynes dismembered pyeloplasty was done and antegrade DJ stent was placed in all cases (Figure-3). We found the advantage of direct access to the UPJ with good exposure of pelvis and renal vessels with this extraperitoneal approach without the need of extending the incision.

### Postoperative follow-up and care

The patient was kept on intravenous fluid till recovery of bowel sounds. Intravenous broad spectrum antibiotic (ceftriaxone) and tramadol on patient demand were administered. Subjective outcome was assessed using visual pain analog scoring (VAS) which was done one day before surgery, on first and second post-operative days and 6 months after surgery in follow-up period. When the drainage tube output was less than 30 mL in 24 hours it was removed in all the patients. The subjects were discharged following Foley catheter and drainage tube removal. The DJ stent was removed after 6 weeks of operation under IV sedation and antibiotic use. Objective parameters were evaluated by an IVP or USG performed at 3 months of follow-up. Subsequent follow-up of patients were done at 6 months and then annually. At each visit, apart from history and clinical examination, serum creatinine and USG were obtained. DTPA scan was done at 6 months and repeated at 1 year of follow-up and yearly afterwards.

### Statistical analysis

The analysis was carried out by using SPSS version 16.0. The results are presented in mean ± standard deviation and percentages. The continuous variables were compared by unpaired t-test and changes in variables from pre-operative to post operatives were compared by paired t-test. The p-value <0.05 was considered as significant.

## RESULTS

The baseline demographic characteristics of patients are shown in Table-1. A total of 71 patients were included. Among them, 17 patients were in pediatric age group (less than 18 years) and the remaining 54 were more than 18 years old. All patients underwent Anderson Hynes dismembered pyeloplasty through muscle-splitting mini-incision approach. Mean incision length was 5.2 cm. The mean operating time was 63 (52-124) minutes and the mean blood loss was 41.23 (27-78) mL (Table-2). None of the patients required blood transfusion. In 27 (38%) patients, anterior crossing vessels at UPJ were encountered and transposed during UPJ reconstruction. Complications were recorded and graded using Dindo-modified Clavien classification of surgical complications (Table-3). The overall complication rate was 8.4%. No major complications occurred during or after the surgery. DJ stent was found to be blocked in one patient with persistent pain and fever with excessive drain output. The patient was managed by replacement of double J catheter. Three patients developed post-operative febrile urinary tract infection, managed by culture specific antibiotics. No patient developed hernia at incision site or other wound related complications. Mean visual pain analog score (VAS) preoperatively was 2.6±0.46; it was 3.8±0.73 and 1.2±0.26 on the first and second post- operative day respectively and 0.27±0.43 at 6 months after surgery. The mean hospital stay in this study was 2.5 (2–6) days (Table-2). The double-J stent was removed 6 weeks postoperatively under local anesthesia and sedation.

**Table 1:** Baseline demographic characteristics of patients.

Total No of patients		71
Mean (range) (years)		23(07-42)
No. M/F		44/27
Mean BMI (kg/m2)		23.6 (18-27)
Mean (range) S. Creatnine		1.1 (0.3-1.5)
No. Rt/Lt side of involvement		23/48
**Symptoms**		
	Lumbar pain	59
	Abdominal mass	10
	Asymptomatic	2
No. of pts with associated stones		16
Horse-shoe kidney with UPJO		2
**No. of pts with**		
	pre-op PCN	4
**No. of pts with co-morbidity**		
	DM	4
	HT	6

**Table 2:** Peri-operative and postoperative parameters.

Mean (range) operative time (mints)	63 (52-124)
Mean±SD VAS pre-operatively	2.6±0.46
Incision length (cm)	5.2 (3.8-7.4)
Mean±SD VAS on day 1	3.8±0.73
Mean±SD VAS on day 2	1.2±0.26
Mean±SD VAS post- operatively at 6 months after surgery	0.27±0.43
No. of pts with anterior crossing vessels	27
Mean (range) blood loss (mL)	41.23(27-78)
Mean (range) days of drain removal	2.1 (2 to 5)
Mean(range) hosp stay (days)	2.5 (2-6)
Median Follow-up (months)	16(9-22)
Overall complications (%)	5(8.4%)
Overall Success rate (%)	98.6

**Table 3:** Post operative complications by Dindo-modified clavien classification of surgical complications.

Clavien Grading	Complication	No. of pts in open group
I	Wound infection	1
II	Febrile UTI	3
IIIa	Displaced stent in post op period	1
IIIb	Re- surgery	1

The overall success rate was 98.6%. In 16 cases, concomitant renal stones were successfully removed and no recurrence of renal calculi was noted till last follow-up. The median follow-up period was 16 months (range 9-22 months). All the patients were symptom free during the follow-up period. Comparison of pre and post-operative parameters are shown in Table-4. Most patients had grade III or IV hydronephrosis on ultrasound and severe to moderate hydronephrosis on IVP before surgical intervention. Post-operative renal ultrasound or IVP demonstrated decrease or complete resolution of hydronephrosis in almost all patients. The follow-up DPTA scan was suggestive of significant improvement in drainage in comparison to previous scans. While preoperative mean T1/2 was 26.7±6.4 minutes, postoperative half-time decreased to 7.8±4.2 minutes at 6 months and to 6.7±3.3 mints at 1-year after surgery. Mean preoperative DRF on DTPA scan was 26.45±10.32% and it was 31.38±8.74% and 33.19±06.13% at 6 months and 1 year respectively.

**Table 4:** Comparison of pre and post operative parameters.

Subjective outcome	Pre-operatively	On post op day 1	6 months after surgery	P value blw pre-op and at 6 months
Pain Score (VAS)	2.6±0.46	3.8±0.73	0.27±0.43	0.0001
**Objective outcome**	**Pre-operatively**	**At 6 months of follow-up**	**At 1 Year Follow-Up**	**P value blw pre-op and at 1 year**
Mean DRF (%)	26.45±10.32	31.38±8.74	33.19±06.13	0.0001
T1/2 (min)	26.7±6.4	7.8±4.2	6.7±3.3	0.0001

HDN Grades on Renal USG	Pre-operatively (no. of pts)	At 6 month follow up (no. of pts)	At 1 year follow up(no. of pts)
Grade IV	59	10	3
Grade III	10	14	2
Grade II	2	17	9
Grade I	—	27	16
No HDN	—	3	41

IVP Grading of HDN	Pre-operatively (no. of pts)	At 6 month follow up (no. of pts)	At 1 year follow-up (no. of pts)
Severe	58	11	2
Moderate	13	17	5
Mild	—	39	13
NO HDN	—	4	51

## DISCUSSION

Open pyeloplasty was originally described by Foley in 1937 and modified by Anderson and Hynes and since then, the open pyeloplasty is considered the gold standard treatment for UPJO with success rates of more than 90% (13).

A significant post-operative pain and a long recovery time with incision site scar are the major disadvantages of the open pyeloplasty. Many minimally invasive approaches were introduced to minimize the drawbacks of open pyeloplasty which include endopyelotomies either by antegrade or retrograde route, acucise endopyelotomy, balloon dilation, laparoscopic pyeloplasty or more recently robotic pyeloplasties (14). By using endoscopy and laparoscopy, the morbidity of surgery can be minimized especially in ablative and reconstructive urologic procedures. However, with the exception of laparoscopic and robotic pyeloplasties, other minimally invasive surgeries present lower success rates (61% to 89%) with significant risk of bleeding compared with open pyeloplasty (15).

Laparoscopic pyeloplasty as a treatment option for the UPJO combines the advantage of an open reconstruction under direct magnified vision with the low morbidity of an endoscopic approach (16, 17). Schuessler et al. described the first transperitoneal access in 1993 (18); the retroperitoneoscopic approach to pyeloplasty was first reported by Janetschek et al. in 1996 (19). Laparoscopic pyeloplasty is reported in several series but transperitoneal route was more commonly employed in those studies (20). Open pyeloplasty is performed by lumbar posterior approach rather than the transperitoneal (a more anatomical direct approach), with better exposition of the renal pelvis (19). In our opinion, the use of laparoscopic techniques should not involve a change in the surgical approach. Also, the steep learning curve has hindered laparoscopic pyeloplasty acceptance into mainstream urological practice (21).

There are few non randomized studies comparing conventional open pyeloplasty with laparoscopic pyeloplasty (22, 23) which seem to favour the latter. However, this comparison studies involved the standard open technique, not the ‘mini’ approach (incision length <8 cm), which results in far less morbidity than the conventional operation. In a study, Chacko et al. concluded that minimal invasive open pyeloplasty is a safe and effective treatment of choice for UPJO in pediatric age group with success rates reaching 95% after surgery (24). Minimally invasive surgery could be performed with a small incision without excessive post-operative analgesic requirement. Sutherland et al. achieved a success rate of 95% in children <1 year and 96% in children >1 year in a study of 234 pediatric pyeloplasties (25). The present cohort study using mini incision open pyeloplasty technique compares favorably with previously reported literature in children and even in adults.

Minimally invasive open pyeloplasty has the distinctive advantages over endourological procedures in terms of overall success rates and in the management of associated crossing vessels. Another issue is that endourological procedures are compromised by varied surgical anatomy of PUJ (high ureteric insertion or huge dilatation). These are also associated with a higher risk of peri-operative hemorrhage with blood transfusions rate of 3-11% (6, 8, 13). In comparison to open or laparoscopic pyeloplasty, success rates of these minimally invasive endoscopic surgeries (Acucise cutting balloon, antegrade percutaneous endopyelotomy, and retrograde endopyelotomy) are approximately 10% to 25% lower and hemorrhagic complications are more frequent (26, 27).

Soulie et al. reported that shorter anterior incision (mean 5 cm) with muscle splitting reduces the risk of chronic pain and wound herniation (28). The results of this study revealed no significant difference between the two techniques (LP and OP with minimal incision) in terms of morbidity, functional results, post-operative pain and return to normal activity. Laparoscopic pyeloplasty was described as a difficult procedure requiring careful ureteral dissection and intra-corporeal suturing.

In a recent meta-Analysis done by Stefanie A et al., re-intervention (RI) rate and re-do-pyeloplasty rate were twice more frequent in laparoscopy group (LP) in comparison to open pyeloplasty (29). The reason for higher RI in LP group was absence of tactile sensation and more tissue trauma at site of anastomosis.

In the present study, we used relatively shorter muscle-splitting incision, thus, avoiding injury to subcostal neurovascular bundle which lies between internal oblique and transversus abdominis muscles to reduce postoperative pain and other complications. As patients of different age groups were included in the present study (both children and adults), mean length of 5.2 cm incision was used (3.8-7.4 cm). While Klingler and Zhang et al. used 23.8±9.1 cm and 21 cm incision respectively in their comparison study of open versus laparoscopic pyeloplasty with consequent abdominal wall herniations and thromboembolism due to long incision and subsequent prolonged hospital stay (2, 3), no such complications occurred in the present study.

Mean operating time was 63 (52-124) minutes in the present series. The mini-incision technique has the definite advantage of reduced overall duration of surgery in comparison to most of published series of laparoscopic pyeloplasty and thus, less anesthesia related complications (30). Mean blood loss in this study was 41.23 (27-78) mL, which is comparable to other laparoscopic and open studies (30).

In the present series, complications were recorded and graded according to Dindo-modified Clavien classification of surgical complications. Overall complication rate was 8.4% which is comparable to those reported in laparoscopic and open literature (30). There was no major complication in our series and most of them were managed conservatively.

In our study, the success rate was 98.6% which is comparable to standard open and laparoscopic pyeloplasty. Most of the patients in present series had BMI less than 25 (mean 23.6); minimal incision approach may not be successful in more obese patients. High success rates are the sheer advantage of mini-incision pyeloplasty over other less invasive techniques. The results of present study clearly indicate that reconstructive urological procedures can effectively be done by mini-incision approach with minimal morbidity and comparable success rates.

Because of smaller operative space, laparoscopic pyeloplasty was found to be technically challenging in children in comparison to adults. So, pediatric population seems to be specially benefitted by mini-incision approach as equal cosmesis can be achieved, avoiding technical difficulty of laparoscopic approach (22). We also observed that mini-incision approach was also feasible in the anomalous kidneys. In the present study, 2 of the 71 patients had a horseshoe kidney with UPJO. In another patient, UPJO was present in solitary functioning kidney. Pyeloplasty was performed in those patients uneventfully.

In the present study, mean visual pain analog score (VAS) pre-operatively was 2.6±0.46. VAS was 0.27±0.43 when measured at 6 months of follow-up period, showing significant decrease of subjective symptoms. The lower pain score and the decreased consumption of postoperative analgesics allow early ambulation and resumption of oral intake.

There was significant improvement in the objective parameters after the surgery in the follow up period. There was either decrease or complete resolution of hydronephrosis on renal ultrasound or IVP which further decreased on serial follow-up scans. There was significant improvement in drainage pattern on follow-up DTPA renal scans in comparison of preoperative scans in the majority of patients. Mean radiotracer wash out time was more than 20 minutes in the majority of the patients pre-operatively. Significant decrease in half time was observed post-operatively at 6 month renal scan and reached a non-obstructed level. Mean differential renal function on follow up DTPA scan showed improvement in differential function in comparison to previous scan.

Thus, mini incision pyeloplasty can easily be adapted by surgeons who are experienced in standard open pyeloplasty. This technique has a shorter hospital stay, early convalescence and better cosmesis. It is cost-effective and is an ideal substitute for the centres where laparoscopy is still evolving.

## CONCLUSIONS

Mini-incision pyeloplasty is a safe and effective technique of open pyeloplasty. It has high success rates similar to open standard pyeloplasty with lower morbidity than laparoscopic approach. Excellent functional and objective outcomes can be achieved if performed by an experienced surgeon.
